# Disability Measurement for Lymphatic Filariasis: A Review of Generic Tools Used within Morbidity Management Programs

**DOI:** 10.1371/journal.pntd.0001768

**Published:** 2012-09-27

**Authors:** Lynne Zeldenryk, Susan Gordon, Marion Gray, Richard Speare, Wayne Melrose

**Affiliations:** 1 School of Public Health, Tropical Medicine and Rehabilitation Sciences, James Cook University, Douglas, Queensland, Australia; 2 Occupational Therapy, University of the Sunshine Coast, Sippy Downs, Queensland, Australia; Centers for Disease Control and Prevention, United States of America

## Abstract

Lymphatic filariasis (LF)-related disability affects 40 million people globally, making LF the leading cause of physical disability in the world. Despite this, there is limited research into how the impacts of LF-related disability are best measured. This article identifies the tools currently being used to measure LF-related disability and reviews their applicability against the known impacts of LF. The findings from the review show that the generic disability tools currently used by LF programs fail to measure the majority of known impacts of LF-related disability. The findings from the review support the development of an LF-specific disability measurement tool and raise doubt about the suitability of generic disability tools to assess disability related to neglected tropical diseases (NTDs) globally.

## Background

Lymphatic filariasis (LF) is caused by three filarial nematodes: *Brugia malayi*, *Brugia timori*, and most commonly, *Wucheria bancrofti* [1]. Globally 120 million people have LF and 1.34 billion people are at risk within endemic regions (65% within South-East Asia, 30% in the African region, and the remaining in other tropical regions) [2].

It is estimated that 40 million people are chronically disabled by LF, making LF the leading cause of physical disability in the world [3]. In the chronic stages, LF can cause severe lymphoedema of limbs and genitalia, scrotal hydrocele, rheumatic, and respiratory problems [4]. Lymphoedema affects approximately 15 million people, whilst scrotal hydrocele affects approximately 25 million men globally [2].

The Global Program to Eliminate Lymphatic Filariasis (GPELF) recently released their progress report for 2000–2009 [2]. The report summarized the work of the GPELF's first decade, which was focused on implementing mass drug administration (MDA) across all LF endemic regions. The report acknowledged that whilst MDA programmes have been particularly successful in reducing infection within communities, efforts to reduce morbidity associated with LF remain lacking. Currently, only 26 of the 81 endemic countries have morbidity programs [2]. These programs focus on hygiene, skin care, hydrocele surgery, and exercises [5]. The GPELF plan for 2010–2020 highlights the need for the establishment of morbidity management programs in all endemic regions. In particular, the plan identifies the need for the development of metrics to monitor and report on the outcomes of these programs [2].

Globally, the approach to disability measurement has shifted over the past two decades. Previously, disability and the impact of disease were conceptualized using a medical model. Measures of mortality, years living with impairment [6], or in the case of LF, the stage and type of impairment [2] reflected a purely medical model to health measurement. However, in recent years, the concept of health and wellness has expanded. Disability is now conceptualised not as the presence of an illness or impairment but rather as the relationship between the disease/illness/impairment, the persons functioning within daily activities/social roles, and the social, cultural, and physical environments that enable or limit an individual's ability to participate fully in his or her community and daily lives [7]. As a result, global disability measurement tools have been developed to measure these broad concepts.

Within the LF community, a number of generic disability tools have been recommended and trialled. These include The International Classification of Functioning, Disability and Health (ICF) [8], the WHO Disability Assessment Schedule (WHODAS) (J.M. Fox, personal communication, 2012) [9], [10], and the WHO Quality of Life Tools (WHOQOL and WHOQOL-Bref) [11]. These generic tools are suggested to be necessary and appropriate measures of disability impact for LF as they have been developed and validated internationally (allowing for multi-country surveys) and would allow comparison of findings against other diseases (allowing for the GPELF to potentially raise the awareness of the impacts of LF globally) [8].

However, others have advocated for the development of an LF-specific disability measurement tool, arguing that an LF-specific tool would be more sensitive to the clinical features of LF and for detecting small changes in function that occur with disease progression [12]. Further, it has been argued that an LF-specific tool would allow greater sensitivity in the assessment of outcomes of GPELF interventions, particularly for patients in chronic stages of the disease where the physical impacts are irreversible and quality of life rather than cure becomes the aim of intervention [11].

In order to determine if current generic tools are suitable for disability measurement within LF programs, this article aims to summarize the reported issues of LF-related disability and review generic tools being used for LF measurement for relevance to these known issues of LF disability.

## Methods

A database search of MEDLINE, CINAHL, Scopus, and ProQuest databases was conducted to identify disability measurement tools that had been used to measure LF-related disability. Keywords used in the search (both separately and in Boolean combination) included but was not limited to: “lymphatic filariasis,” “elephantiasis,” “lymphoedema,” “lymphocele,” “hydrocele,” “disability,” “rehabilitation,” “morbidity,” “evaluation,” “measurement,” “assessment,” “monitoring,” “severity of illness,” “examination,” and “health screening.” Grey literature was also reviewed. Inclusion criteria were as follows: (a) the tools measured the experience of disability from a patient perspective and (b) the tools measured the lived experience or impacts of LF-related disability rather than solely the physical or medical attributes of the disease. Exclusion criteria were as follows: (a) tools that measured the clinicians' perspective of disability rather than the patients', (b) tools that measured solely medical issues of LF-related disability rather than the impact/experience of living with LF-related disability, and (c) survey instruments were specific to study rather than psychometrically tested generic disability tools for generic disability measurement. From the search, 12 studies were identified that had used generic disability measures, of which five were excluded as they were non-standardized, self-developed research questionnaires rather than psychometrically tested generic disability instruments.

The questions included in each tool used for LF measurement were reviewed against the key issues of LF-related disability as reported in the literature (see Table 1). These known issues are taken from a previous critical review of the qualitative research into the experiences of people living with LF-related disability [13]. Issues of LF-related disability were included in the review if they were found to be central to the experience of LF-related disability in two or more studies. Key issues that were only identified within one study were not included in the review, as it was hypothesized that these issues may have been relevant to the context of the single study, rather than a key issue of LF-related disability within other regions/populations. Content analysis of the key issues was then completed by the research team to identify broad themes, referred to as “domains” within this study.

**Table 1:** Matrix of domains and issues of LF-related disability as identified in the literature.

Domains	Issues	Ahorlu 1999 [28]	Ahorlu 2001 [29]	Babu 2009 [30]	Bandyopadhyay 1996 [31]	Coreil 1998 [32]	Gyapong 2000 [33]	Perera 2007 [34]	Person 2006 [35]	Person 2007 [36]	Person 2008 [37]	Person 2009 [38]	Suma 2003 [39]	Total
Clinical Manifestation; Hydrocele, H, Lymphoedema, L		H	H, L	H	L	L	H	L	L	L	L	L	L	
Psychological	Depression					√		√	√	√	√	√	√	7
	Feelings of shame/humiliation	√	√					√			√	√	√	6
	Low self-esteem/inferiority		√		√							√		3
	Feeling unattractive/poor body image				√				√	√	√			3
	Ability to cope/strategies					√		√			√	√		4
	Grief/loss of former self								√	√		√		2
	Fear		√		√		√				√	√	√	6
	Wishing they were dead										√	√		2
	Embarrassment				√	√		√	√			√		5
	Feeling isolated					√		√			√			3
	Hopelessness								√			√		2
	Frustration			√									√	2
	Feeling inadequate								√					1
	Feeling like a burden						√							1
Activities	Sexual functioning	√	√	√			√							4
	Work	√	√	√	√	√	√	√			√		√	9
	Mobility		√			√							√	3
	Childcare				√			√						2
	Domestic chores				√	√							√	3
	Catch transport/cycling etc.					√							√	2
	Work agitates condition						√	√					√	3
	Self-care					√							√	2
	Sleep					√								1
Participation	Marriageability			√		√	√							
	Personal relationships	√	√	√	√	√	√		√			√	√	9
	Attend social events	√	√			√			√		√	√	√	7
	Ability to go to school				√	√		√	√					4
Environmental	Teasing	√	√			√					√	√		5
	Avoided by others				√						√	√	√	4
	LF reduced social status	√		√	√			√				√	√	6
	Stigma within family		√						√		√	√	√	5
	Stigma within community	√	√	√		√		√	√		√	√	√	8
	Families as carers				√	√		√						3
	Child generates income							√						1
	Treatment availability					√	√	√						3
	Expense of treatment		√					√	√					3
	Location of treatment							√						1
	Stigma within health system							√				√		2
	Stigma within school system											√		1
	Appropriate foot care					√								1
	Access to support groups					√			√		√			3
	Hygienic home conditions							√						1
	Hygienic work conditions							√						1
Personal	Poverty			√				√	√			√		4
	Education status				√				√			√		3
	Pain			√		√	√	√			√			5

## Findings

### Issues of LF-Related Disability

The most commonly reported issues relevant to LF-related disability were impact of LF on work (nine studies), stigma surrounding LF within local communities (nine studies), impact on personal relationships (nine studies), depression (seven studies), impact of LF on social events (seven studies), feelings of shame/humiliation experienced by LF patients (six studies), fear (six studies), and reduced social status (six studies) (see Table 1). Least commonly reported issues (reported in one study only) relevant to LF-related disability were feelings of inadequacy, feeling like a burden, sleeping problems, location of treatment, stigma within the school system, access to appropriate foot-care, un-hygienic home conditions, and un-hygienic work conditions.

### Disability Measurement Tools Currently Used for LF Morbidity Measurement

Three tools have been previously used to measure LF-related disability: The seven domains five levels (7D5L) instrument, which is a seven-item, extended form of the European Quality of Life Instrument (EuroQol 5D3L) [14], [15]; The Dermatology Life Quality Index (DLQI) [16]–[19], a 10-item tool designed to measure the impact of skin disease on quality of life (QOL); and The ICF checklist [8], a multi-item checklist based on the WHO ICF social model of health [8], [20].

Another three tools have either been advocated for use in LF measurements or have been used for LF disability surveys but have not been formally published [9]. These are the WHO Quality of Life tool (WHOQOL), a hundred-item QOL measurement [11], [21]; the WHOQOL Bref [11], [22], which is a shortened (26-item) version of the WHOQOL tool; and the WHO Disability Assessment Schedule (WHODAS) [9], [10], [23], a 36-item disability measurement tool.

### Relevance of Generic Tools to LF-Related Disability Issues

The 34 issues reported by people living with LF-related disability were compared against the items from each of the six disability measurement tools (see Table 2). Some items within the tools measure broad concepts (i.e., “usual activity” item of the 7D5L tool) that relate to a range of issues within the activity domain (however did not specifically represent any of the separate issues). Of the 34 issues reported to be relevant for people living with LF, 11 would not be identified by any of the current measurement tools (feelings of shame/humiliation, low self-esteem/feeling of inferiority, ability of cope, grief, fear, frustration, work agitates condition, teasing by others, avoided by others, families becoming carers, and lack of access to support groups).

**Table 2:** Items of generic tools as they relate to domains and issues of LF-related disability.

Domain	Items	7D5L [40]	DLQI [41]	WHODAS II 36 [42]	ICF Checklist [43]	WHOQOL-100 [44]	WHOQOL BREF [45]
Psychological	Depression	Anxiety/depression item					
	Feelings of shame/humiliation						
	Low self-esteem/inferiority						
	Feeling unattractive/poor body image					Items 9, 30, 41	Item 11
	Ability to cope/strategies						
	Grief/loss of former self						
	Fear						
	Embarrassment		Item 2				
	Feeling isolated					Item 32	
	Frustration						
	General items			D6.5 Emotions & Flashcard #1	b152 Emotions		26 negative feelings
	% of psychological domain covered	10%	10%	0%	0%	20%	10%
Daily Activities	Sexual functioning		Item 9	Item D4.5	Item b640	Items 33, 34	Item 21
	Work	Usual activity item	Item 7	Items A5, D5.7–5.12, D5.14	Item d850	Items 89–91	Item 18
	Mobility	Mobility item		Items D2.1–D2.5	Items d4, b710, S720–s760	Items 93–96	Item 15
	Childcare	Usual activity item		Item D5.1			
	Domestic chores	Usual activity item	Item 3	Items D5.1–D5.6	Item d6		
	Catch transport/cycling etc.	Mobility item			Item d470	Item 23	Item 25
	Work agitates condition						
	Self-care	Self-care item		Items D3.1–D3.4	Item d5		
	General items					12 routine activities	17 daily activities
	% of activity domain covered	75%	37.5%	75%	75%	50%	50%
Participation	Personal relationships	Social participation item	Item 8	Items D4.1–D4.4	Items d7 (7 items)	Items 44–45	Items 20,22
	Attend social events	Social participation item	Item 5	Items D6.1–D6.2	Item d910		
	Ability to go to school		Item 7	Items D7.5–D5.12	Item d820		
	% of participation domain covered	66.60%	100%	100%	100%	33.30%	33.30%
Environmental factors	Teasing						
	Avoided by others						
	LF reduced social status				Item e460		
	Stigma within family			Item D6.3	Item e410		
	Stigma within community			Item D6.3	Item e460		
	Families as carers						
	Treatment availability				Item e580		
	Expense of treatment			Item D6.6	Item e580		
	Stigma within health system				Item e450		
	Access to support groups						
	General items			Item D6.7		38 medical access, 44 support from others	
	% of environmental domain covered	0%	0%	30%	60%	10%	0%
Personal	Poverty			Item D6.6, D5.13	Item d870	Items 19, 47	Item 12
	Education status			Item A3	Item a5		
	Pain	Pain/discomfort item	Item 1	Flashcard #1	Item b280	Items 2, 25	Item 3
	% of personal domain covered	33.30%	33.30%	100%	100%	66.60%	66.60%
	Total # of LF issues covered by tool	10	8	17	18	10	8
	% of LF issues covered by tool	29%	24%	47%	50%	28%	22%

Content analysis revealed five broad domains that the issues encompassed. These were psychological impacts, impact on daily activities, impact on participation, and the influence of environmental factors and personal factors. In total, the domains that would most comprehensively be assessed by the tools items were found to be daily activities (tools covered 37.5%–75% of issues), participation (tools covered 33%–100% of issues), and personal factors (tools covered 33%–100% of issues). More poorly measured by the tools were psychological issues (tools covered 0%–20% of issues) and environmental factors (tools covered 0%–60% of issues) relevant to LF-related disability.

The ICF checklist was found to have items that captured the most issues (50%), followed by the WHODAS 36 (47%). However, neither of these tools included any items that would identify the 10 specific issues included within the psychological domain. The other tools had very few items that would identify issues related to LF disability: 7D5L (29%), WHO-QOL 100 (28%), DLQI (24%), and WHOQOL Bref (22%).

## Discussion

The research that informs our knowledge of the impact of LF-related disability is limited and still emerging [13]. However, key issues of LF-related disability have been found across studies within multiple countries, suggesting that these issues are relevant and common to LF patients globally. The findings reveal that the greatest number of issues/impacts of LF-related disability falls within the environmental and psychological domains—two areas of need where GPELF has failed to develop intervention strategies. Whilst the GPELF continues to identify prevention and alleviation of disability as a key second pillar of the program [24], there is limited movement and financial support within the program to develop substantial rehabilitation programmes that (a) support mental health and well-being, (b) minimize barriers from stigma through advocacy work, (c) provide adequate intervention for those living with chronic LF to prevent further disease progression, and (d) assist in re-engagement with daily activities and life roles that are important for patients both physically, mentally, and socially.

This review identified that the tools currently being used to measure LF-related disability are inadequate. This review revealed 34 issues across five domains that are consistently reported by people living with LF-related disability. Of the six measurement tools (four generic, two specific) that have been used to measure LF-related disability, only one measurement tool (ICF Checklist [8]) included 50% of relevant issues, whilst others covered between 22% and 47% of the known issues of LF-related disability. Hence, current disability measurement tools used within the field fail to measure at least half of the known impacts of LF-related disability.

Importantly, the majority of tools do not measure the most commonly reported issues of LF-related disability. The most commonly reported psychological issues, feelings of shame/humiliation, low self-esteem, and fear are not measured by any tools. Likewise, the most commonly reported environmental issues are not well measured by the tools; teasing is not measured by any tool, whilst the impact on social status is captured by one tool and stigma within family and stigma within communities are measured within two tools. However, impact of LF on work and personal relationships, two of the most commonly reported impacts of LF, are measured by all tools.

Whilst generic disability tools have been developed to capture social and functional impacts of disease, the tools reviewed in this article were inadequate to measure the majority of the known impacts of LF. Indeed other authors have reported that generic tools often do not capture disease-specific aspects and are insensitive to detecting key changes in patient status making them poor outcome measurement tools for disease-specific studies [25]–[27]. Generic tools, such as the ICF, WHOQOL, and WHODAS, whilst useful for comparison studies between diseases, will not effectively measure the impacts of LF and outcomes of GPELF programs. If they are the only tools used by public health planners to capture the impact of LF, they are likely to underestimate the true impacts of LF globally and be poor measures of the success of GPELF programs within LF endemic regions.

## Conclusion

The ability to measure LF disability progression and the impact of interventions over time in a standardized manner is essential for the GPELF. The development of an LF-specific disability assessment tool, relevant for LF impact and the contexts and cultures of LF endemic areas, is vital for accurate GPELF reporting and measurement. A focus on the second pillar of the GPELF program, morbidity management, is increasingly required as MDA programs finish. Valid and reliable information about patient and community needs and the measurement of outcomes of the second pillar of the GPELF program are required to ensure best management for the prevention and alleviation of LF-related disability.

**Key Learning Points**

1. Current generic disability measurement tools used within the LF field fail to adequately measure the known impacts of LF-related disability.
2. In particular, current tools fail to adequately measure psychological and environmental factors relevant to the lived experience of LF-related disability.
3. There remains a need for an LF-specific quality of life/disability measurement tool to be developed to adequately measure the impact of LF-related disability for individuals and communities.

**5 Key Papers in the Field**

1. Kumari AK, Krishnamoorthy K, Harichandrakumar K, Das L (2007) Health Related Quality of Life, an appropriate indicator to assess the impact of morbidity management and disability prevention activities towards elimination of lymphatic filariasis. Filaria J 6: 8.
2. Babu B, Nayak A, Rath K, Kerketta A (2006) Use of the dermatology quality of life index in filarial lymphoedema patients. Trans Roy Soc Trop Med Hyg 100: 258–263.
3. Chandrasena T, Premaratna R, Muthugala M, Pathmeswaran A, de Silva N (2007) Modified Dermatology Life Quality Index as a measure of quality of life in patients with filarial lymphoedema. Trans Roy Soc Trop Med Hyg 101: 245–249.
4. Harichandrakumar K, Krishnamoorthy K, Kumari A, Das L (2006) Health status of lymphatic filariasis assessed from patients using seven domains five levels (7D5L) instrument. Acta Trop 99: 137–143.
5. McPherson T (2003) Impact on the quality of life of lymphoedema patients following introduction of a hygiene and skin care regimen in a Guyanese community endemic for lymphatic filariasis: a preliminary clinical intervention study. Filaria J 2: 1.
